# Microbiome and transcriptome analyses reveal the influence of calcined dolomite application on *Eriocheir sinensis* in a rice–crab co-culture system

**DOI:** 10.1038/s41598-023-39099-1

**Published:** 2023-10-20

**Authors:** Yingdong Li, Lishong Li, Wei Miao, Xiaodong Li

**Affiliations:** College of Animal Science and Veterinary Medicine, Shenyang Agricultural University, Dongling Road 120, Shenyang, 110866 China

**Keywords:** Biochemistry, Ecology, Microbiology, Environmental sciences

## Abstract

Co-culture systems of rice and aquatic animals can contribute to the ecological intensification of agriculture by reducing nutrient loss and the need for N fertilizer application and by enhancing nutrient-use efficiency. However, the input of high-protein diets into paddy fields, to facilitate the growth of aquatic animals, has been found to increase N pollution and acidification of the soil. Although soil amendments have been widely used to ameliorate acidic soils, reduce N_2_O emissions, and improve agronomic production, the relationship between soil amendments and aquatic animal remains unclear. Therefore, this study investigated the effects of calcined dolomite (hereafter referred to as dolomite) as an acidic soil amendment and Ca–Mg supplement in rice–crab co-culture using *Eriocheir sinensis* crabs (Chinese mitten crabs). High-throughput sequencing was used to examine crab bacterial community composition and crab hepatopancreas biology. Although the water pH was significantly increased in the dolomite group, the number, composition, and diversity of bacteria identified in crab gut microbiome did not vary significantly between the dolomite and control groups. In the dolomite group, the probiotic agents *Candidatus Hepatoplasma* and *Lactobacillus* were highly abundant in the crab gut, and immune- and retinol metabolism-related genes were significantly upregulated in the crab hepatopancreas. Overall, dolomite application increased crab health and water pH. Dolomite is a low-cost amendment, with better stability, compared to other soil amendments, thus making it ideal for sustainable and clean rice–aquatic animal co-culture.

## Introduction

Soil amendments are widely used in agriculture to retain nutrients and pesticides and reduce the contamination of surrounding areas and groundwater^1^. They have positive effects on soil fertility and yields, helping to close nutrient cycles and ensuring food security^2–4^. It is well established that co-culture systems of rice and aquatic animals can contribute to the ecological intensification of agriculture by providing multiple ecosystem services, promoting biological pest control, reducing pesticide use, improving soil quality, and enhancing crop yields^5,6^. Although rice–aquatic animal co-culture can reduce the need for N fertilizer application, enhance nutrient-use efficiency, and reduce nutrient loss, it can also cause severe N pollution and soil acidification as aquatic animals require high-protein diets and excrete N-rich urine and feces into the bottom soil of paddies^7,8^. Nonetheless, the relationship between soil amendments and aquatic animal remains to be clarified.

Soil amendments such as biochar and limestone are widely used in agriculture to address soil acidification and increase soil pH. They have positive effects on soil fertility and yields, helping to close nutrient cycles and ensuring food security^2,3^. The use of biochar as a soil amendment positively impacts an array of soil processes, benefiting soil biology, controlling soil-borne pathogens, enhancing N fixation, improving soil physical and chemical properties, decreasing NO_3_^−^ leaching and N_2_O emissions, and remediating contaminated soils^1^. However, limestone is the most widely applied amendment in agricultural soil because it has a lower price and application dosage than biochar^9^. Moreover, compared to biochar, liming can increase the concentrations of Ca^2+^ and exchangeable Mg^2+^ and soil cation exchange capacity and reduce potential acidity (H^+^  + Al^3+^)^10^.

Among the available amendments, dolomitic lime has been verified as an effective amendment for paddy field soil contaminated by trace elements^11^. As it is relatively inexpensive and simultaneously provides Ca and Mg, dolomite has been widely used in agriculture in China in the recent years^12,13^. Calcined dolomite is better than uncalcined dolomite in improving soil acidity^14^. After dolomite is calcined, the CaCO_3_ and MgCO_3_ in the ore are transformed into CaO and MgO, which react with soil water to produce Mg(OH)_2_ and Ca(OH)_2_. In addition, Mg(OH)_2_ and Ca(OH)_2_ can gradually release calcium and magnesium while neutralizing soil acidity, providing calcium and magnesium nutrients for the paddy field.

Rice–crab co-culture is a rice-based ecological aquaculture system commonly used in China^15,16^. It improves soil nutrient levels and increases soil-to-rice nutrient translocation capacity^7^. However, economically important aquatic animals, such as fish, crayfish, and frogs, require high-protein diets for gonad development and weight gain^17,18^. Inevitably, the input of high-protein diets into paddy fields results in increased N pollution and acidification of the soil. Our previously published study indicated that high-protein diets can reduce soil pH and microbial diversity in rice–crab co-culture^8^. Thus, this study aimed to investigate the effects of dolomite, a type of limestone that is common and affordable in China, on paddy field water quality, the abundance and community composition of microbes in *Eriocheir sinensis* crabs (Chinese mitten crabs), and the physiological status of the crabs when applied in a rice–crab co-culture environment. We hypothesized that dolomite amendment could increase Ca^2+^ and Mg^2+^ supply and have desirable effects on the physiological status of crabs. Thus, by determining the efficiency of soil amendments in rice–crab co-culture systems, we aim to provide key solutions for improving food production while promoting environmental safety.

## Materials and methods

### Dolomite amendment material and effects on water quality

Calcined dolomite, with a composition was 35.70% MgO, 32.76% CaO, 0.92% Fe_2_O_3_, 0.78% SiO_2_, and 0.2% Al_2_O_3_, was purchased from Dashiqiao Jiali Refractory Company (Yingkou City, Liaoning Province, China) in May 2021.

Before the paddy field experiment, 5 dolomite concentrations were tested for their impact on water: 0 kg/m^2^ (A), 0.5 kg/m^2^ (B), 1.5 kg/m^2^ (C), 2.5 kg/m^2^ (D), and 3.5 kg/m^2^ (E). Each concentration had 3 repeats, and each repeat used a self-made container (w × l × h = 40 × 50 × 20 cm) filled with 20 L of tap water. Water quality parameters were monitored every 3 days for 1 month at the Chemical Analysis Laboratory of the Shenyang Agriculture University. Temperature and pH values were tested using a pen-like pH meter (pH-100A; Shanghai Lichen Co., Ltd., Shanghai, China), dissolved oxygen (DO) was measured using a portable dissolved oxygen meter (PB-607A; Shanghai Leici Co., Ltd, Shanghai, China), and the salinity was determined directly with a pen-like salinity meter (AR-8012A; Smart Sensor Co., Ltd., Guangdong, China). Alkalinity, hardness, ammonia–nitrogen (NH_3_–N), and nitrate–nitrogen (NO_2_–N) were followed standard procedures described in APHA^19^.

### Field description and experimental design

The experiments were performed in 6 paddy fields (each 17 × 10 m) at Panjin Guanghe Crab Industry Co., Ltd., Panjin, Liaoning Province, China, during the 2021 rice-growing season (over 5 months, from 1st, May to 10th October). In total, 12,000 late megalopa-stage *Eriocheir sinensis* crabs (Chinese mitten crabs; average weight, 7.34 ± 1.32 mg) were obtained from Panjin Guanghe Crab Industry and randomly distributed within the 6 fields (2000 crabs/field). The two treatments in the field experiment were set as no dolomite treatment (CK) and dolomite treatment (DOL). Based on the results of our previous experiment, the dolomite quantity in the DOL group was set at 1.5 kg/m^2^. During the 5-month experiment, crabs were fed commercial diets (Well Hope Foods Co., Ltd., China, containing 40% protein) at 3% of their body weight per day.

### Sample collection

To investigate the transcriptome and 16S rRNA changes in crabs induced by dolomite, 15 healthy crabs were randomly collected from each of the CK and DOL treatment groups on October 10, 2021. The hepatopancreas and intestines of each crab were extracted and placed in separate 2-mL RNase-free tubes. They were immediately frozen in liquid nitrogen until RNA and DNA extraction. All sampling was performed on a sterilized workbench.

### Metagenome analysis of crab intestine microbial community structure

#### DNA extraction and PCR amplification

Total genomic DNA was extracted from the crab intestine samples using an OMEGA Soil DNA kit (Omega Bio-Tek, Norcross, GA, USA) according to the manufacturer’s instructions, and stored at − 20 °C for further analysis. The quantity and quality of the extracted DNA were evaluated using a NanoDrop ND-1000 spectrophotometer (Thermo Fisher Scientific, Waltham, MA, USA) and agarose gel electrophoresis, respectively. Briefly, the V3–V4 region of the bacterial 16S ribosomal RNA gene was amplified using PCR with the primers F 5′-ACTCCTACGGGAGGCAGCA-3′ and R 5′-CGGACTACHVGGGTWTCTAAT-3′. The forward primer ITSF (5′-GGAAGTAAAAGTCGTAACAAGG-3′) and reverse primer ITSR (5′-GCTGCGTTCTTCATCGATGC-3′) were used to amplify the fungal internal transcribed spacer (ITS) V1 regions^20^. Thermal cycling consisted of initial denaturation at 98 °C for 5 min, followed by 28 cycles of denaturation at 98 °C for 30 s, annealing at 55 °C for 30 s, and extension at 72 °C for 45 s, with a final extension of 5 min at 72 °C. Amplicons were extracted from 2% agarose gels and purified, using Vazyme VAHTS DNA Clean Beads (Vazyme, Nanjing, China) according to the manufacturer’s instructions, and then quantified using a Quant-iT PicoGreen dsDNA Assay Kit (Invitrogen, Carlsbad, CA, USA). The diversity and composition of the microbial communities in the soil and crab intestine samples were analyzed based on the raw sequencing data obtained using the Illumina MiSeq platform at Shanghai Personal Biotechnology Co., Ltd. (Shanghai, China), according to standard protocols.

#### Bioinformatics and statistical analysis

After sequencing, raw FASTQ data were processed using QIIME2 and R v. 3.2.0, with slight modifications based on official tutorials (https://docs.qiime2.org/2019.4/tutorials/). According our previously published methods, amplicon sequencing variant (ASV)-level alpha diversity indexes, taxonomic composition and abundance, relative abundance, hierarchically clustered and heat map analysis was used to identify high-dimensional biomarker taxa with significantly different abundances among the DOL and CK groups^8^. Heat maps were generated using the heatmap.2 function from the gplots R package, based on Spearman correlation matrices (without q-value thresholding). The raw reads have been deposited with the NCBI (BioProject numbers PRJNA788377).

### Transcriptome sequencing of crab hepatopancreas

#### cDNA library preparation and transcriptome sequencing

Total RNA was extracted from the crab hepatopancreas using Trizol Reagent (Invitrogen Life Technologies), after which, RNA concentration, quality, and integrity were determined using a NanoDrop spectrophotometer (Thermo Fisher Scientific). mRNA was purified from total RNA that had been predigested at 37 °C for 1 h using DNase I and a Micropoly (A) PuristTM mRNA purification kit (Ambion, Austin, TX, USA). Equal amounts of mRNA samples from five crabs were pooled to generate one mixed sample, and three biological repetitions were conducted for both CK and DOL groups. A separate Illumina sequencing library was prepared using 10 µg of mRNA per sample.

#### De novo assembly and gene annotation

The original FASTQ data were filtered, and reads with connectors, lengths less than 50 bp, and an average sequence quality less than Q20 were removed. The high-quality sequences were spliced from scratch to obtain transcripts. These transcripts were clustered, and the longest transcripts were selected as unigenes^21^. The gene function of each unigene was annotated. The databases used for gene function annotation included NR (https://ftp.ncbi.nlm.nih.gov/blast/db/FASTA/), GO (http://geneontology.org/), KEGG (https://www.kegg.jp/), eggNOG (http://eggnog5.embl.de/), Swiss-Prot (http://www.gpmaw.com/html/swiss-prot.html), and Pfam (http://pfam.xfam.org/).

### Statistical analyses

Significant differences at the 95% level were identified by one-way ANOVA followed by Tukey’s multiple range test using SPSS software (v. 17.0; IBM Corp., Armonk, NY, USA). To identify differentially expressed genes (DEGs) across samples or groups, edgeR (v.3.2) was used. The DEGs were then subjected to enrichment analysis of GO functions and KEGG pathways. The related analyses were performed using the “corrgram” package in the R environment (R v. 3.2.0). The data were plotted using Origin v. 9.0 (OriginLab, Northampton, MA, USA).

### Ethical approval

Our study did not involve endangered or protected species. In China, the breeding and capture of Chinese mitten crabs (*Eriocheir sinensis*) and collecting rice plant in rice fields do not require specific permits. All efforts were made to minimize animal suffering and discomfort. The experimental protocol was approved by the Animal Ethics Committee of Shenyang Agriculture University (permit number 2021041601). This study is reported in accordance to ARRIVE guidelines (https://arriveguidelines.org/arrive-guidelines). All animal and plant surveys were carried out in accordance with the approved guidelines of Shenyang Agriculture University Experimental Animal Management Committee.

## Results

### Impact of dolomite on water quality

Figure S1 presents the water temperature, DO, pH, salinity, alkalinity, hardness, NH_3_–N content, and NO_2_–N content of water in the laboratory and field simulations of the rice field system treated with different quantities of dolomite. During the 30-day experiments, no significant differences in temperature, salinity, NH_3_–N, or NO_2_–N were found among the 5 groups (P > 0.05). The pH of dolomite-treated water in all groups was significantly higher than that of the control group (P < 0.05), increasing gradually with increased amounts of dolomite. DO was not conspicuously altered in the high-dolomite groups (2.5 kg/m^2^ and 3.5 kg/m^2^). However, with 0.5 kg/m^2^ of dolomite, DO was significantly reduced compared to the other groups (P < 0.05). Alkalinity in the 3.5 kg/m^2^ group increased significantly after 3 days compared to the other groups.

### Impact of dolomite on crab intestine bacterial diversity

**Figure 1:**
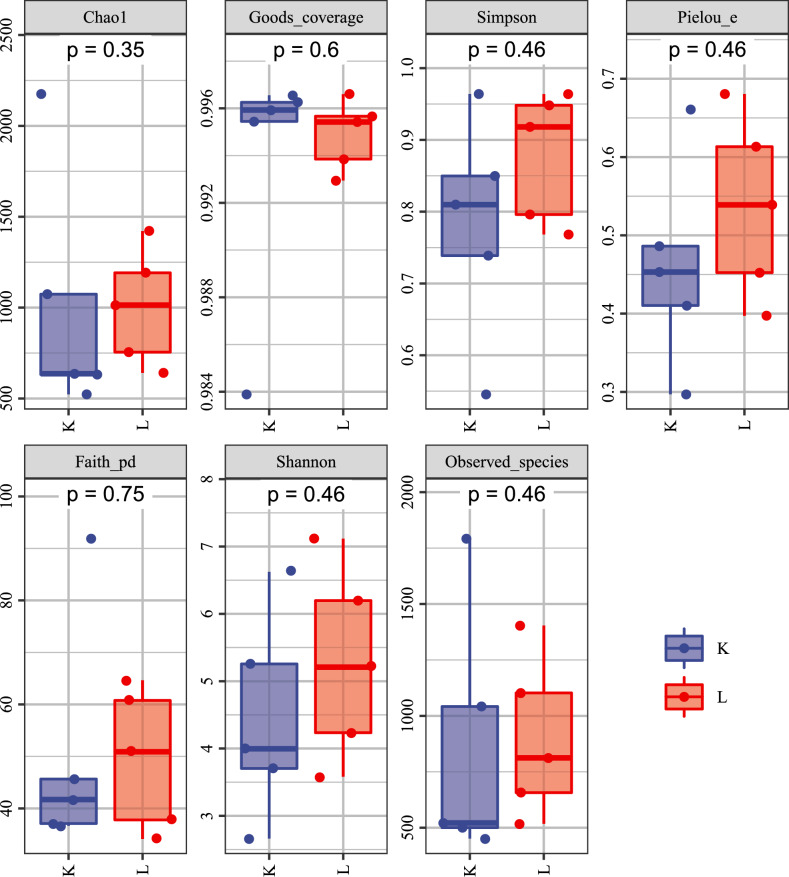
Alpha diversity of bacterial groups in crab gut from the dolomite and control groups in a rice–crab co-culture system.

A total of 868,803 raw sequence reads were generated from the gut samples. After quality filtering and denoising, 786,111 clean reads were retained, with an average of 63,959 non-singleton reads per sample (Table 1). We used alpha diversity indices to estimate bacterial richness and diversity in the DOL and CK groups. Chao1, Shannon, and Simpson indices did not differ significantly between the DOL and CK groups (Fig. 1). This finding was supported by the rarefaction and rank abundance curves, which illustrate the richness and diversity of each sample, respectively (Figs. S2, S3). Beta diversity was evaluated using principal coordinates analysis and non-metric multidimensional scaling (Figs. S4, S5).

**Table 1:** Sequences data for bacterial of crab guts in crab rice coculture system collected in July 2021 and October 2021.

Crab gut samples	Bacterial					
	Input	Filtered	Denoised	Merged	Non-chimeric	Non-singleton
Control						
L_1	82,017	75,605	74,006	72,473	62,469	62,216
L_2	104,365	97,345	95,656	93,687	85,331	84,885
L_3	65,756	60,696	58,521	55,172	37,470	36,705
L_4	73,702	68,003	66,255	64,179	49,421	48,810
L_5	98,018	91,391	90,162	88,995	80,498	80,314
Dolomites						
K_1	101,591	94,163	92,330	90,704	84,701	84,381
K_2	88,211	81,651	79,822	78,386	69,924	69,691
K_3	99,155	92,649	89,078	83,095	75,866	74,546
K_4	74,412	67,880	65,882	63,632	40,510	39,957
K_5	81,576	75,852	74,399	73,089	58,331	58,089

**Figure 2:**
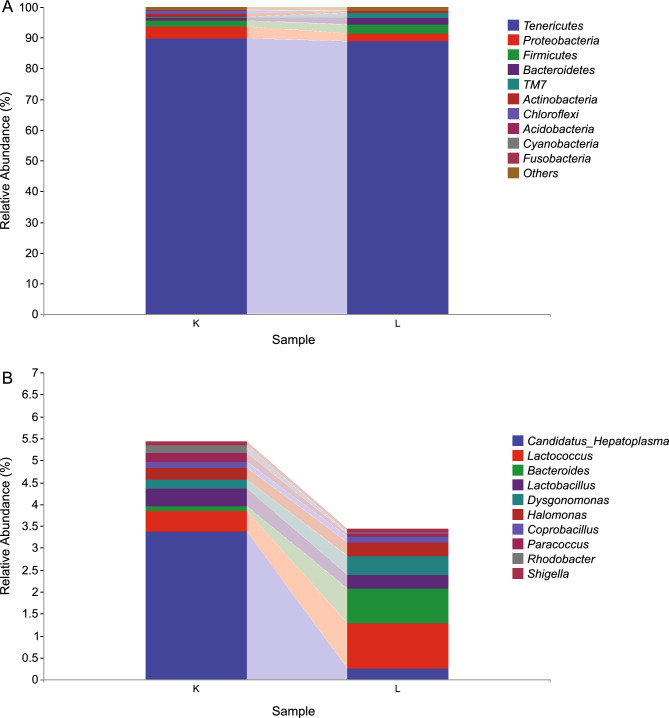
Relative abundance of phyla (**A**) and genus (**B**) in crab gut from the dolomite and control groups in a rice–crab co-culture system.

The five most abundant gut bacterial phyla were Tenericutes, Proteobacteria, Firmicutes, Bacteroidetes, and TM7 (Fig. 2A). Dolomite had no effect on taxonomic composition at the phylum level but caused significant differences at the genus level. As shown in Fig. 2B, the probiotic bacterium *Candidatus Hepatoplasma* was more abundant in the dolomite group, while acidophilic bacteria such as Lactococcus and Bacteroides were more abundant in the control group.

**Figure 3:**
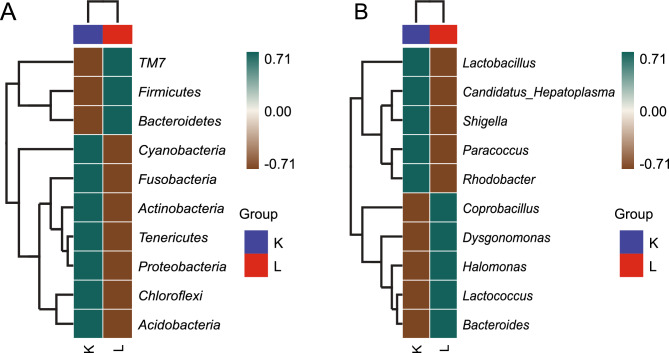
Hierarchical clustering analysis of phyla (**A**) and genus (**B**) in crab gut from the dolomite (L) and control groups (K) in a rice–crab co-culture system.

The heat map showing correlations of phylum-level abundance within different time points revealed that TM7, Firmicutes, and Bacteroidetes were highly abundant in the control group (Fig. 3A). However, at the genus level, Lactobacillus, C. Hepatoplasma, Shigella, Paracoccus, and Rhodobacter were more abundant in the dolomite group than in controls (Fig. 3B). These patterns further demonstrate that probiotic bacteria populations were significantly higher in the dolomite group than in the control group.

### Crab hepatopancreas MiSeq gene expression analyses

**Figure 4:**
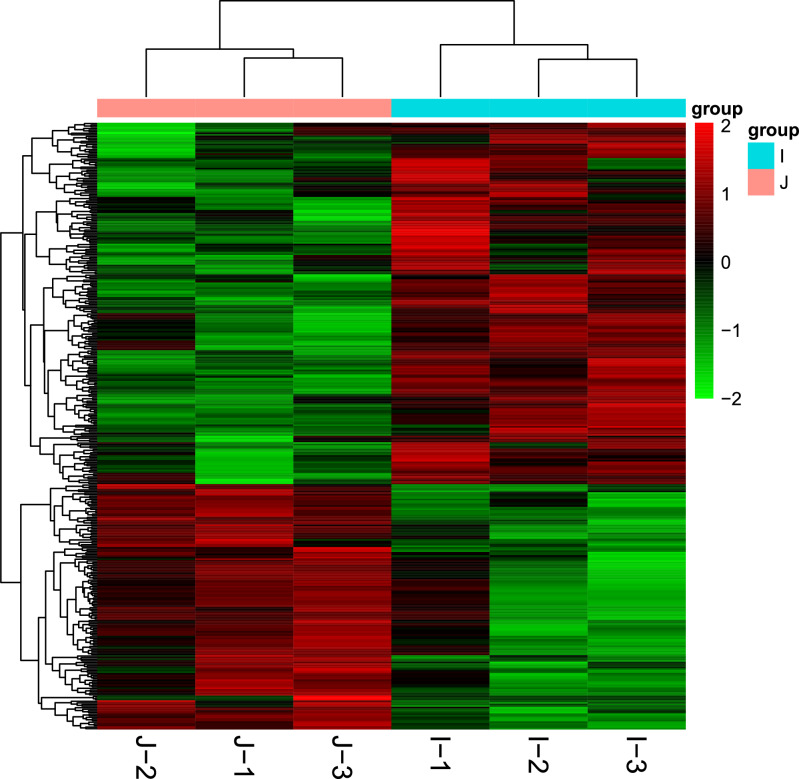
Heatmap clustering of differentially expressed genes (DEGs) from the transcriptomes of the dolomite and control groups. Heatmap was generated using R package “gplots” v3.1.3 (https://github.com/talgalili/gplots).

A summary of the transcriptomic sequences is presented in Table 2. In the NR database, 90.92% of unigenes were annotated (Table 3). Compared with the control group, the dolomite group showed 376 DEGs (P < 0.05), with 224 upregulated genes and 152 downregulated genes (Table S1). Heat map clustering results also indicated that most DEGs could be divided into two classes (Fig. 4).

**Table 2:** Summary of the transcriptomic sequences in control group and dolomites group.

Sample	Clean Reads No	Clean Data (bp)	Clean Reads %	Clean Data %	NCBI Accession Number
Control-1	40,898,788	6,175,716,988	92.26	92.26	SRR17267136
Control -2	44,438,120	6,710,156,120	92.39	92.39	SRR17267135
Control -3	43,862,312	6,623,209,112	93.01	93.01	SRR17267134
Dolomites -1	40,344,172	6,091,969,972	92.85	92.85	SRR17267133
Dolomites -2	37,023,412	5,590,535,212	92.82	92.82	SRR17267132
Dolomites -3	41,870,232	6,322,405,032	92.12	92.12	SRR17267131

**Table 3:** Annotation in database.

Annotation in Database	Unigene NO	Percentage (%)
NR	25,489	90.92
GO	8363	29.83
KEGG	13,332	47.55
Ensembl	28,033	100
eggNOG	23,395	83.45
Swissprot	20,799	74.12

**Figure 5:**
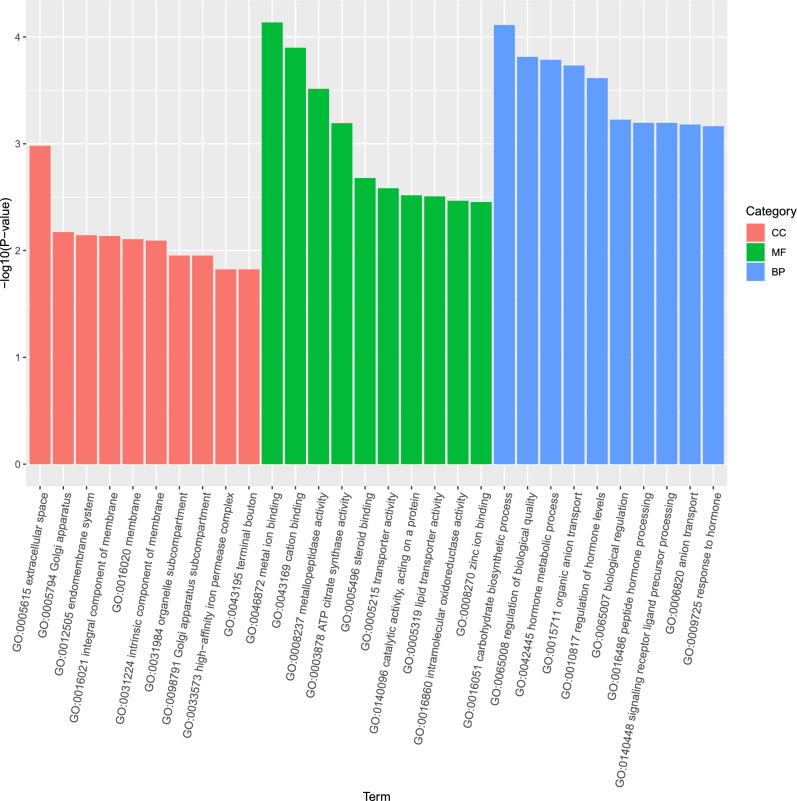
GO annotation of assembled genes.

The results of GO analysis showed that the top three pathways under the “cellular component” category were “metal ion binding”, “cation binding”, and “metallopeptidase activity” (Figure S4). A directed acyclic graph of the “cellular component” category was constructed (Fig. 5), which indicated that metal binding-related pathways were enriched. Using KEGG enrichment analysis, the most significantly enriched metabolism-related pathway was found to be “fructose and mannose metabolism” followed by “retinol metabolism”, which was predominantly enriched in the upregulated DEGs (Figure S6).

After the uncharacterized genes among the 376 DEGs were eliminated between the control and dolomite groups, we obtained 157 upregulated genes (Table S2) and 118 downregulated genes (Table S3). The DEGs related to immune function, including glutathione peroxidase, catalase, cytochrome P450, cryptocyanin, heat shock protein (HSP) 70, and HSP 90, were significantly upregulated in the dolomite group. Moreover, four retinol dehydrogenase (RDH) genes and three ecdysteroid-related genes were also upregulated.

## Discussion

Rice–fishery co-culture can reduce the need for chemical fertilizers, enhance land productivity, and maintain soil fertility^16,22,23^. Although the excreta of aquatic animals provides sufficient nutrients for crop growth, our previous research showed that the high-protein diets of these animals negatively affect paddy soil and cause acidification^8^. Moreover, the main cause of soil acidification in China is the accumulation of exogenous protons and the loss of basic ions such as Ca^2+^ and Mg^2+^ caused by the excessive application of N fertilizer^24^.

Lime is commonly applied to soil to correct soil acidity^25^. Available liming materials include limestone (CaCO_3_), quick lime (CaO), slaked lime [Ca(OH)_2_], and dolomite [CaMg(CO_3_)_2_], which vary in their ability for neutralizing acidified soil. Among these, dolomite, which is rich in Ca and Mg, has been demonstrated to be a better acidic soil conditioner than is lime; moreover, it is cost effective compared to lime^26,27^. After dolomite is calcined, its Ca^2+^ and Mg^2+^ content is gradually released, and the release rate steadily increases over time, which is in line with the growth rhythm^28^. In the present study, calcined dolomite addition increased water pH slowly but steadily, maintaining a consistent pH of 8.0–8.3 for 30 days; it was not affected by other water chemical indices. With the same application time and amount, quick lime and hydrated lime treatments are more efficient than is dolomite in increasing pH^29^. Nevertheless, based on the results of soil and water pH indices, the application of calcined dolomite tends to be more reliable and economical in terms of effectiveness and stability in minimizing the soil acidity problem in rice–crab co-culture. Moreover, dolomite contains Mg, which is essential for crustacean growth and development^30^.

Chinese mitten crabs are highly valued and are thus an important part of the rice–crab co-culture system. Therefore, it is necessary to understand their physiological status within this system, especially under dolomite application. The number of bacteria identified in the gut microbiome barely varied between the dolomite and control groups, with the results of alpha and beta diversity analysis showing insignificant differences in the composition of the gut bacterial communities. The probiotic bacterium C. Hepatoplasma was more abundant in the dolomite group than in the control group, but the converse was true for the acidophilic bacteria Lactococcus and Bacteroides. C. Hepatoplasma in the intestines of juvenile *Panulirus ornatus* and mitten crabs may play a symbiotic role in nutrient absorption^31,32^. Moreover, Lactobacillus, which has potential probiotic properties in crab^33^, was also more abundant in the dolomite group than in the control group. These findings suggest that the microbial communities in the intestines of crabs have an overall high average similarity among the dolomite and control groups, as reflected by the biodiversity and richness of intestinal microbiota and the most dominant community members at the phylum level.

Notably, Notably, in addition to the findings mentioned earlier, we observed significant upregulation of immune function-related genes, such as glutathione peroxidase, catalase, cytochrome P450, cryptocyanin, HSP 70, and HSP 90, in the hepatopancreas of crabs in the DOL groups compared to the control group. This finding aligns with previous studies in crustaceans, where immune genes are known to be stimulated in response to various stimuli. Conversely, under acidic conditions, these immune genes are typically downregulated^34^. This suggests that maintaining an adequate level of magnesium (Mg) in the environment may play a crucial role in preventing rapid bacterial proliferation and reaching infectious levels in the giant freshwater prawn (*Macrobrachium rosenbergii*)^35^. Over 500 mg/kg MgO supplementation resulted in the best survival and growth of M. rosenbergii post-larvae^36^. In the present study, calcined dolomite had an MgO content of 35.70%, which may also promote growth in mitten crabs.

Based on KEGG pathway analysis, the retinol metabolic pathway and related genes were significantly upregulated in dolomite crabs compared to controls. Retinol affects development through a series of enzymes that control a two-step metabolic pathway. In this metabolic pathway, retinol is first oxidized to retinaldehyde, and retinaldehyde is then oxidized to retinoic acid. Retinoic acid acts as a ligand for retinoic acid signaling activities to directly regulate gene expression. RDH is an important enzyme in retinol metabolism and can protect cells from non-aldehyde-related toxicity. In the present study, the expression of RDH-related genes was significantly upregulated, indicating that dolomite induced retinol metabolism. Lower pH and acidified environments can have a deleterious effect on the overall homeostasis of retinoic acid in cells^37^. Therefore, dolomite application helps to increase water and soil pH and eventually has a positive impact on crab health in rice–crab co-culture systems.

## Conclusions

In recent years, rice–crab co-culture systems have promoted rural revitalization, poverty alleviation, and high-quality production. High-protein diets are widely used in this system, increasing the risk of N pollution and soil acidification due to undigested protein-rich feed and feces. A convenient, low-cost, and stable amendment was applied to regulate agriculture processes, while at the same time increasing the production of rice–crab co-culture systems. This study demonstrated that the addition of calcined dolomite in a rice–crab co-culture system increases water pH and crab health over the whole production period. The present study was conducted only in north of China; therefore, further research is needed to determine the effects of dolomite addition on co-culture systems with different types of soils, particularly under acidic and metal-contaminated field conditions.

### Supplementary Information

Supplementary Figure 1.

Supplementary Figure 2.

Supplementary Figure 3.

Supplementary Figure 4.

Supplementary Figure 5.

Supplementary Figure 6.

Supplementary Legends.

Supplementary Table 1.

Supplementary Table 2.

Supplementary Table 3.

## Data Availability

The raw reads have been deposited with the NCBI (BioSample number SAMN23968654 and SAMN24175538).
