# Supplementary figures and images for: Microbiome and transcriptome analyses reveal the influence of calcined dolomite application on Eriocheir sinensis in a rice–crab co-culture system
Source: Sci Rep. 2023 Oct 20;13:17932. doi: 10.1038/s41598-023-39099-1 (PMC10589332; doi:10.1038/s41598-023-39099-1)

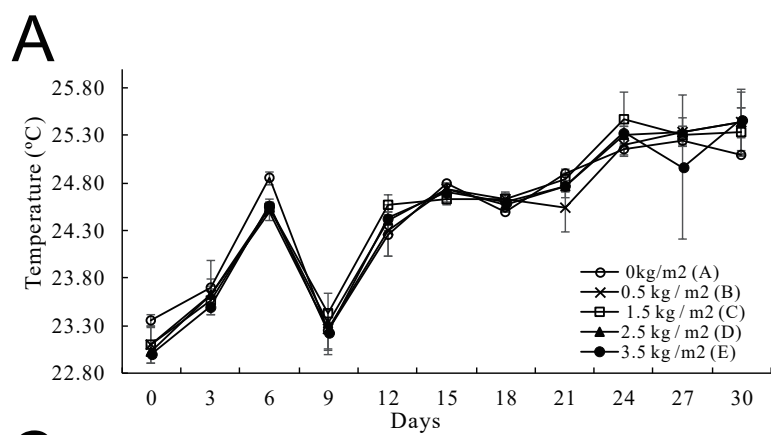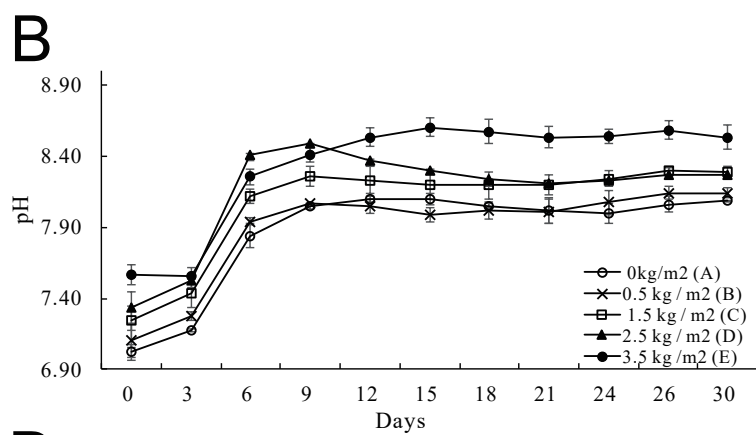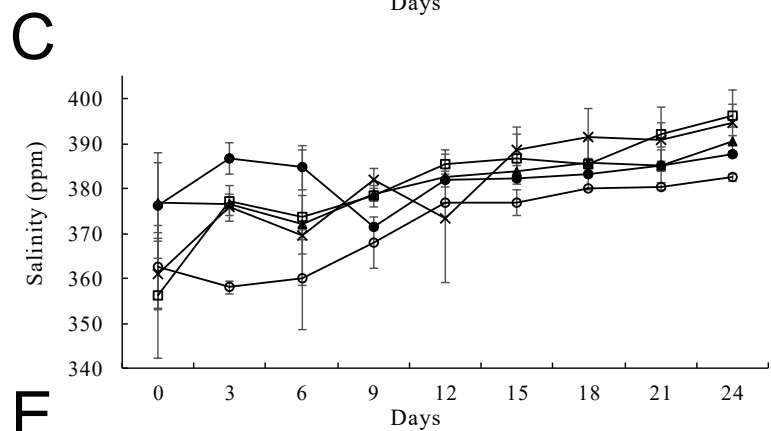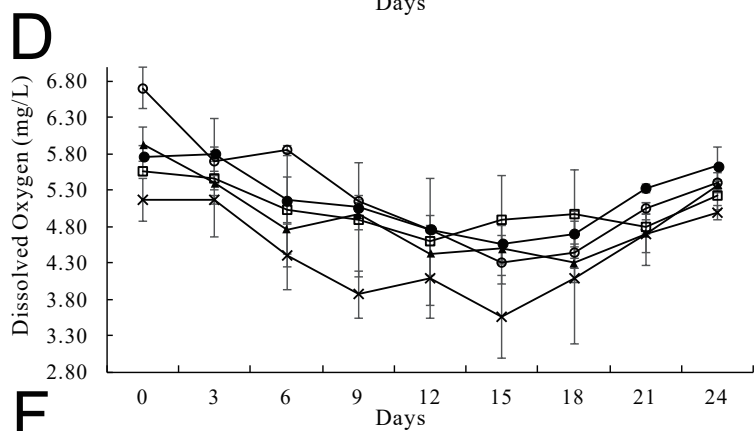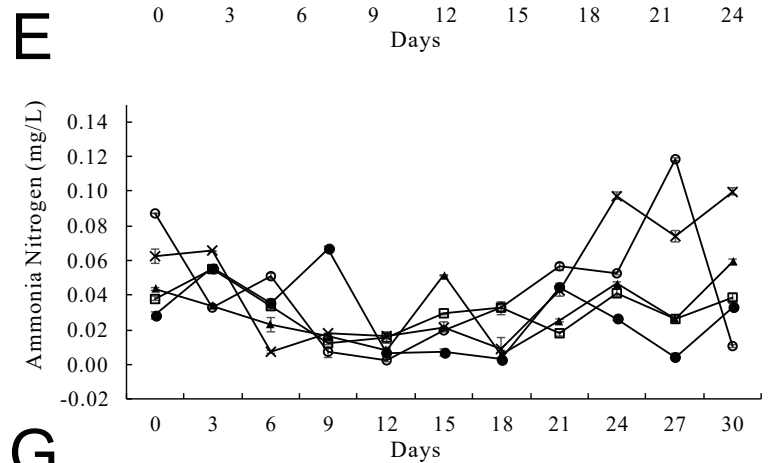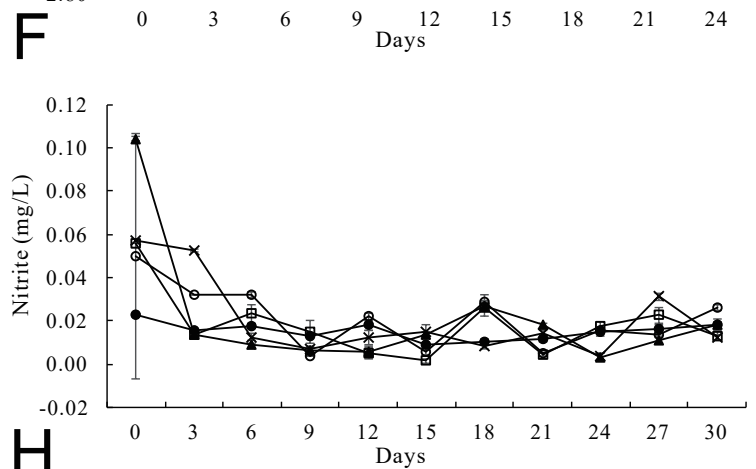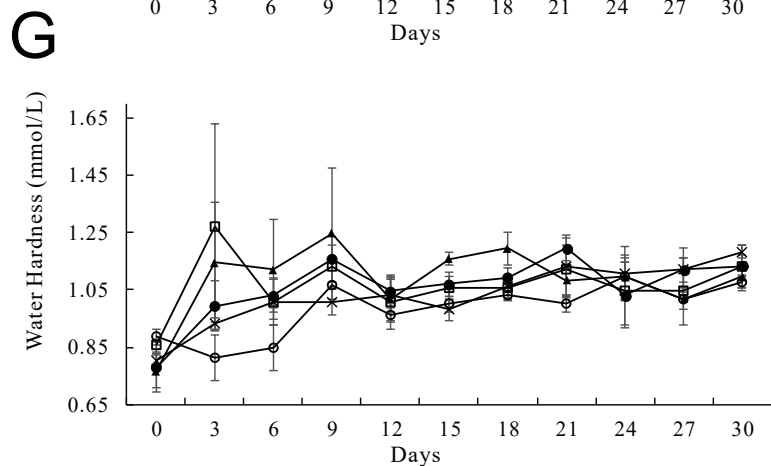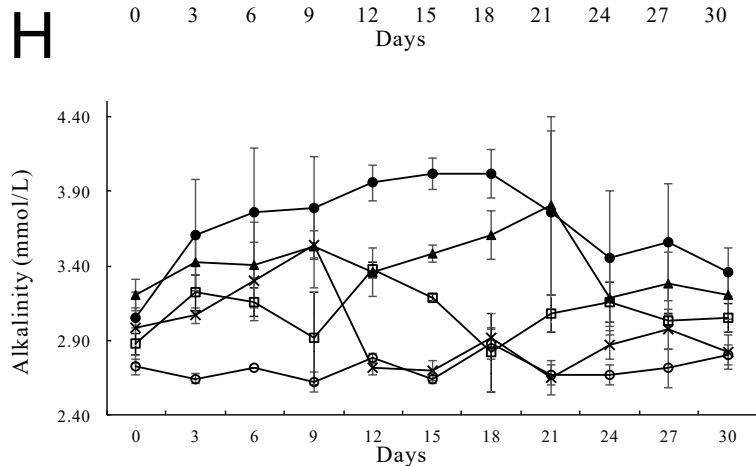

Supplement: Supplementary file 1 — Supplementary Figure 1. [file 41598_2023_39099_MOESM1_ESM.pdf]

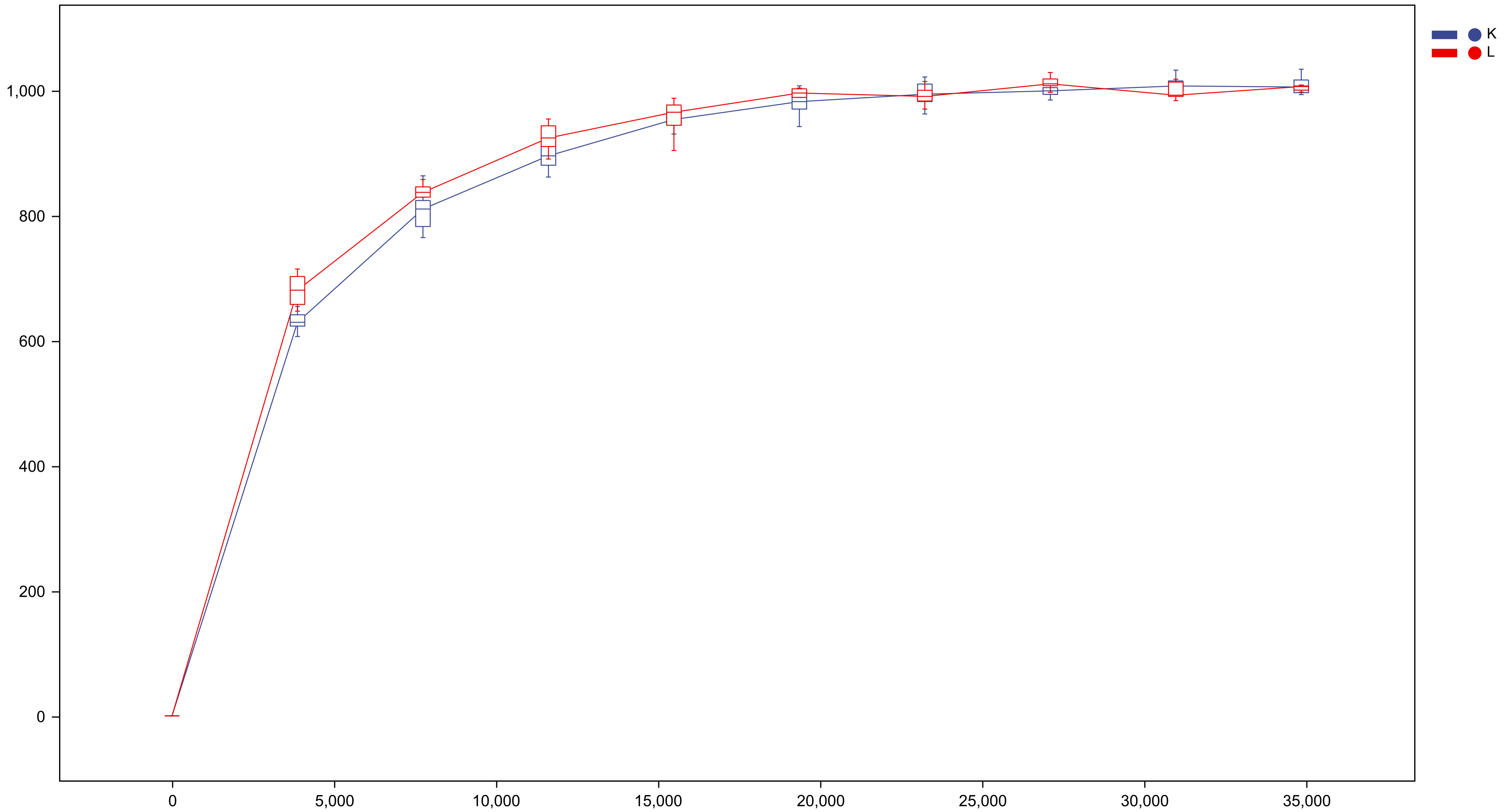

Supplement: Supplementary file 2 — Supplementary Figure 2. [file 41598_2023_39099_MOESM2_ESM.pdf]

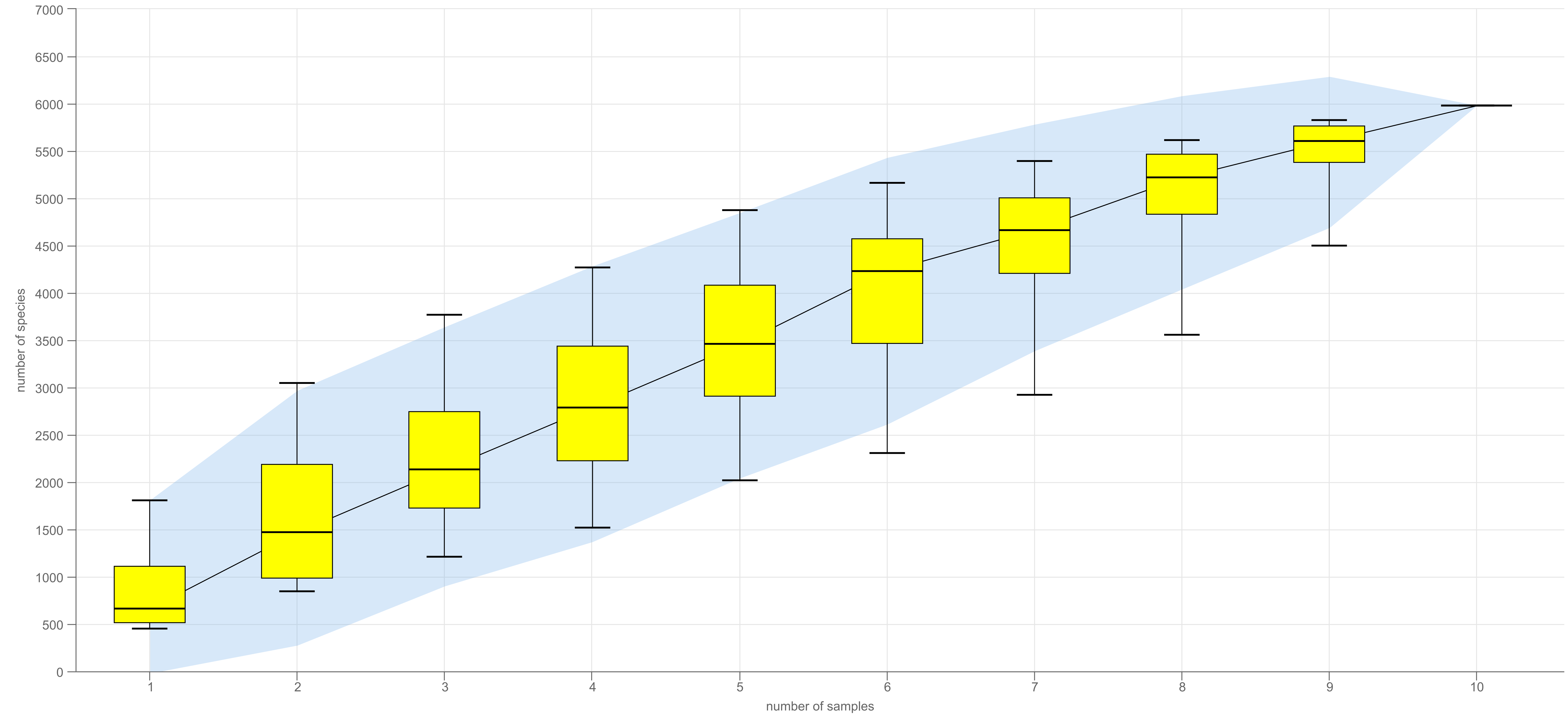

Supplement: Supplementary file 3 — Supplementary Figure 3. [file 41598_2023_39099_MOESM3_ESM.pdf]

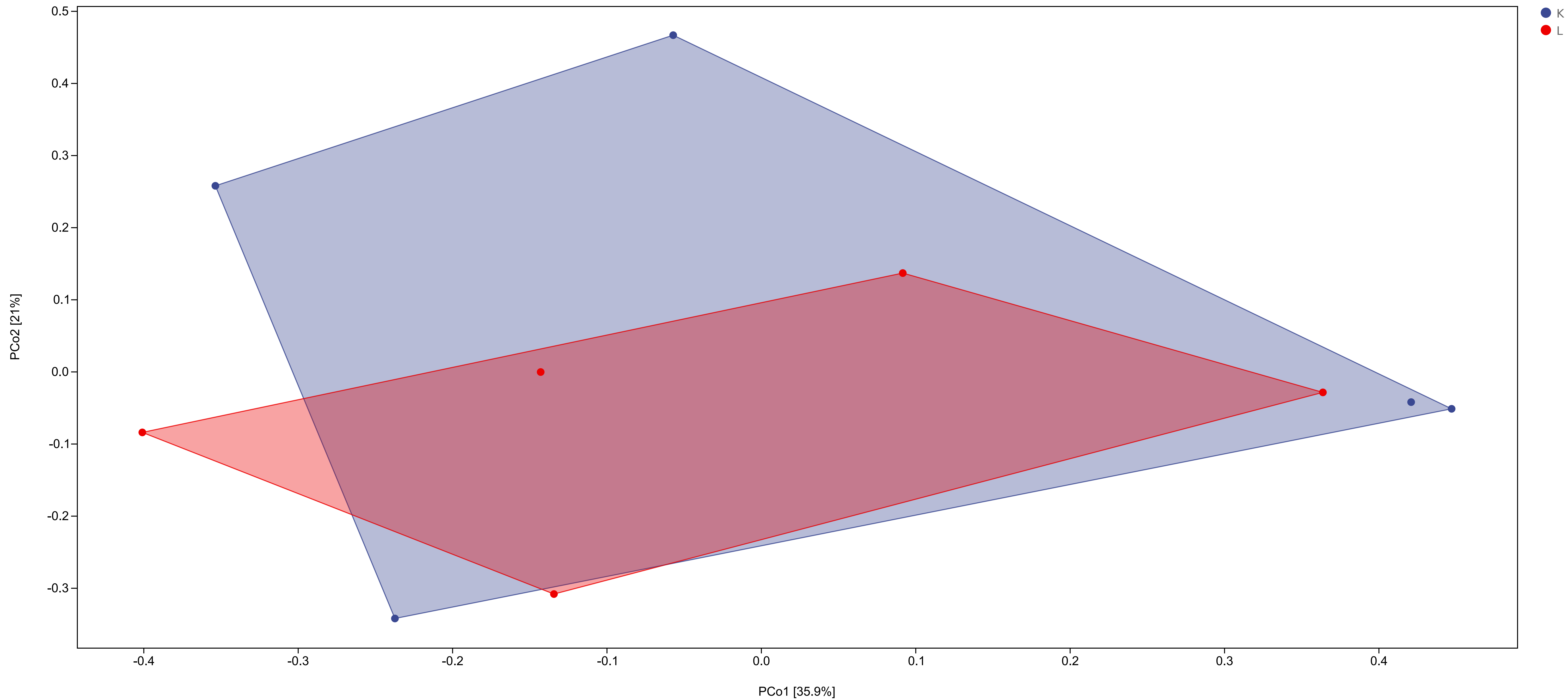

Supplement: Supplementary file 4 — Supplementary Figure 4. [file 41598_2023_39099_MOESM4_ESM.pdf]

Stress = 0.144

● K  
● L

NMDS2

NMDS1

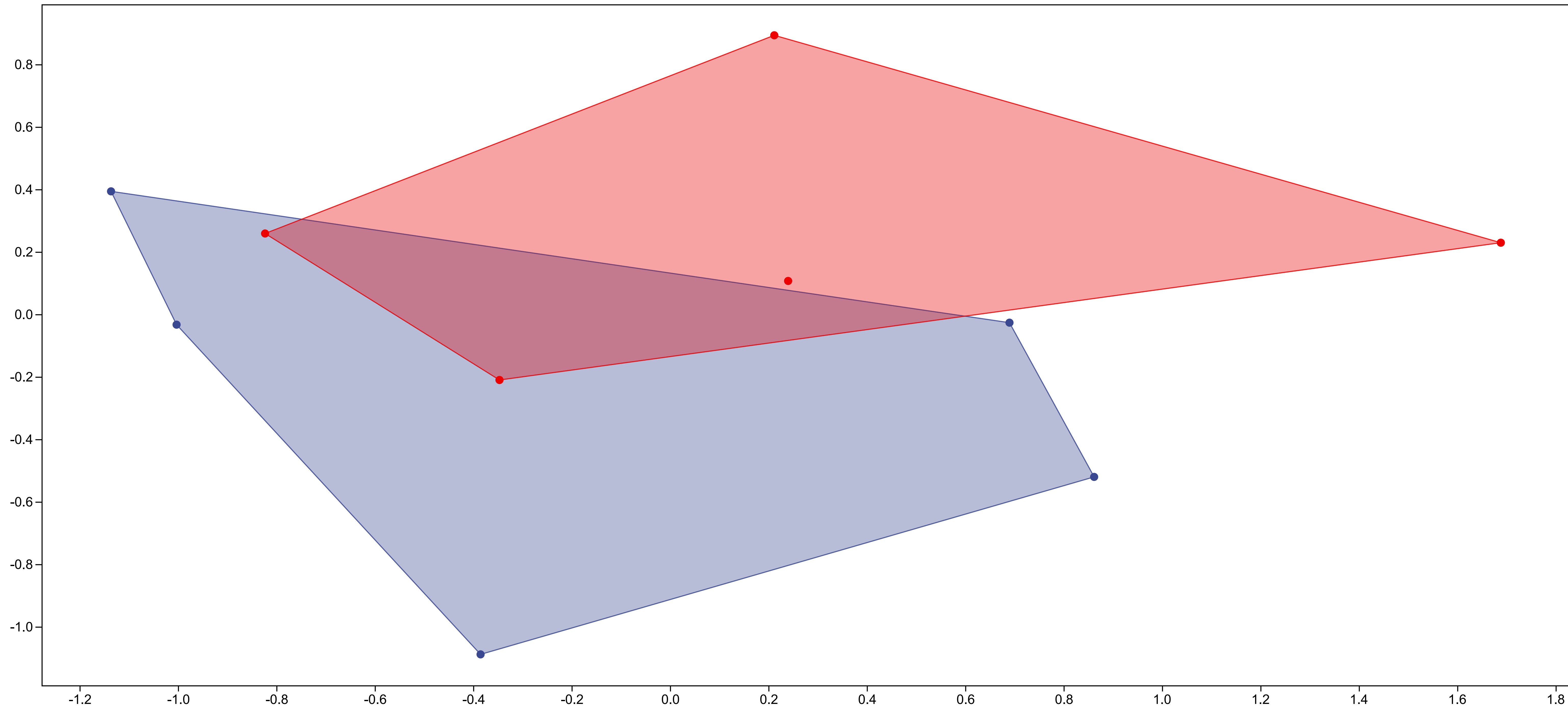

Supplement: Supplementary file 5 — Supplementary Figure 5. [file 41598_2023_39099_MOESM5_ESM.pdf]

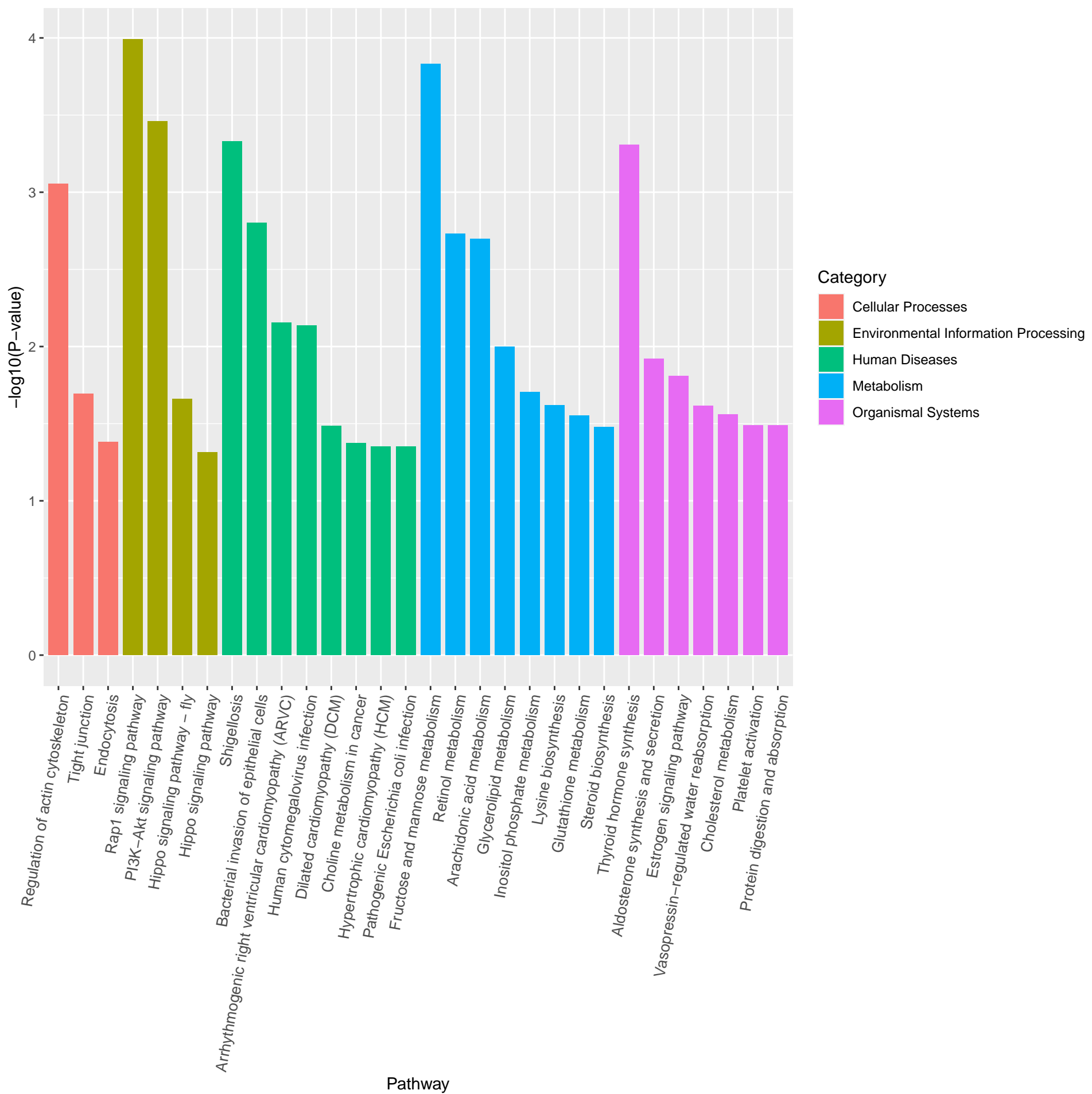

Supplement: Supplementary file 6 — Supplementary Figure 6. [file 41598_2023_39099_MOESM6_ESM.pdf]
