# Supplementary material for: Microbiome and transcriptome analyses reveal the influence of calcined dolomite application on Eriocheir sinensis in a rice–crab co-culture system
Source: Sci Rep. 2023 Oct 20;13:17932. doi: 10.1038/s41598-023-39099-1 (PMC10589332; doi:10.1038/s41598-023-39099-1)
Supplement: Supplementary file 7 — Supplementary Legends. [file 41598_2023_39099_MOESM7_ESM.docx]

**Supplement figure captions**

Figure S1 Water-quality indexes of temperature (A), pH (B), salinity (C), dissolved oxygen (D), ammonia-nitrogen (E), nitrite (F), water hardness (G), and alkalinity (H) from the dolomite and control groups in a rice–crab co-culture system.

Figure S2 Rarefaction curves of bacterial groups in crab gut from the dolomite and control groups in a rice–crab co-culture system.

Figure S3 Rank abundance curves of bacterial groups in crab gut from the dolomite and control groups in a rice–crab co-culture system.

Figure S4 Principal coordinate analysis (PCoA) of crab gut bacterial communities using weighted UniFrac distance metrics.

Figure S5 Non-metric multidimensional scaling (NMDS) ordination plot of crab gut communities based on the number of OTUs detected by pyrosequencing.

Figure S6 KEGG annotation of assembled genes.
